# EHMTI-0336. Metabolic diet therapy in the prophylactic treatment of migraine headache in adolescents by using ketogenic diet

**DOI:** 10.1186/1129-2377-15-S1-G9

**Published:** 2014-09-18

**Authors:** MK Farkas, E Mak, E Richter, V Farkas

**Affiliations:** 1 Dept. of Pediatrics, Semmelweis University, Budapest, Hungary; 2 Dept. of Dietetics and Nutrition Sciences, Semmelweis University, Budapest, Hungary

## Introduction

The high-fat, low-carbohydrate KD is a well-known add-on treatment option for children with medically intractable epilepsy. Anticonvulsants as topiramate can be helpful for migraine prophylaxis in chilndren, however, there are severe cautions by using chronic drug treatment by the parents. Several mechanics may exist for the effect of KD in CNS diseases: including disruption of glutamatergic synaptic transmission, inhibition of glycosis, activation of ATP-sensitive potassium channels and alteration of the mTOR pathway by modifying the brain excitability.

## Aims

The objective of our study was to evaluate the efficacy and tolerability of Ketogenic Diet (KD) in the prophylactic treatment of migraine in adolescents.

## Patient and methods

We have selected adolescents patients between the age of 12-17 years over a 3 month period beginning January 2013. The subjects had a minimum of two attacks per month before applying the ketogenic diet, for at least 1 year. Migraine was diagnosed by IHS-R criteria. A total of 16 children were recruited including 10 females and 6 males. Migraine attack frequency prior to diet and 1, 2 and 3 months after the initiation of KD were compared and the clinical effect of the diet was evaluated. The efficacy was evaluated as completely headache free, markedly effective (attack reduction >75%), effective (reduction >50%) or invalid (reduction <50%). The ratio of fat to carbohydrate plus protein was 3:1. Urine ketones were checked randomly at least twice a week. The KD was started in outpatient care without using a fasting period. The diet was planned for a minimum period of 3 months with the assistance of dieticians.

## Results

Of the total 16 patients 7 discontinued the restrictive diet because of poor compliance (5) and gastrointestinal side effects (2). 9 patients (100%) continued the study for more than 1 month. From those 3 patients were interpreted as invalid responder and discontinued the diet because of lack of efficacy prior to the 2 months visit. 6 subjects (77%) completed the 3 months study period. At the end of the study no subject became completely attack free. The diet was markedly effective for 3 patients (33%) and effective for 3 patients (33%). During the 3 months period an obvious upper trend in the efficacy was observed.

## Conclusions

The compliance of adolescents migraineurs with a restrictive type of KD is problematic, however at the beginning all of the subjects were absolutely open for a non-pharmacological “natural” treatment. Furtheron, most of the patients were additionally motivated to use the KD by its potential weight loss effect, however, all of subjects had clinically normal weight at baseline. An obvious improvement in the headache frequency was observed in about 77% of the patients who were able to continue the restrictive diet for at least a 3 months period. The increase of the efficacy correlated with the duration of therapy.

No conflict of interest.

